# Laceration of the Cornea of Both Eyes, during a Convulsion

**Published:** 1856-07

**Authors:** J. W. McKinney

**Affiliations:** New Albany, Ill.


ARTICLE II.—*Laceration of the Cornea of Both Eyes, during a Convulsion.* Reported by J. W. McKinney, M.D., of New Albany, Ill.

The following unusual case came under my observation recently, a brief outline of which I send you; and you are at liberty to dispose of it as you may think best.

The patient was a child, three years old, step-daughter to Robert Richey. For eight or ten days preceding my first visit, the child had suffered from tertian intermittent, of a mild form, scarcely attracting the attention of the parents—not sufficiently so to resort to medication. On the day previous to my being called, it had a light chill, followed by an exacerbation, which passed off in a few hours, and in a short time it seemed as cheerful as usual.

*Saturday Morning, April 19th,* 1856.—I was summoned by the step-father to see the case, who stated, that the child, soon after awaking from sleep in the morning, was attacked with convulsions. On visiting the patient, I found it lying on the bed with its head and shoulders elevated by means of pillows, and in a state of deep coma. The eyes were closed, pulse quick and frequent, breathing somewhat labored, head hot, while the general heat of the surface was little, if any, above that of the natural temperature. Slight spasmodic jerking of the upper extremities still were present, which readily subsided on the application of cold water to the head, with mustard drafts to the wrists and ankles.

There was nothing peculiar in the symptoms, beyond that of ordinary convulsions of children, to attract my attention, except the coma, which I thought to be of a deeper lethargic character than was usual in such cases. I accordingly ordered a continuation of refrigerants to the head and drafts to the extremities, so long as the head remained preternaturally hot; and prescribed a brisk cathartic of calomel, 10 grs.; turpentine, thirty drops; mixed with a dessert-spoonful of castor oil; to be administered so soon as the patient was sufficiently recovered to swallow.

On the morning of the 20th, when again visiting the patient, I learned that the cathartic dose had been given, which acted freely during the night, and that there had been no return of convulsions; but that the deep coma had continued, and it was with difficulty the patient could be aroused so as to swallow anything when put into its mouth.

On raising the upper lids of the eyes, (which had been kept constantly closed since the convulsion the previous morning,) I discovered a laceration of the cornea of both eyes.

The lacerations were both transverse, pointing to the outer and inner canthus, and just below the lower border of the pupils, presenting smooth edges as though they had been cut by a sharp instrument. The rent in the left eye, extended entirely across the cornea; while that of the right, extended from the inner border of the cornea, or that next the greater canthus, only about two-thirds across its diameter, in the direction just stated.

The eyes were slightly flattened in front, from the escape of a portion of the aqueous humor through the rent, which imparted a dim contracted appearance to the sound portions of the cornea.

Acute inflammation of the conjunctiva of the left eye was set up, radiating from the two extreme points of the laceration.

Together with all this, there was complete paralysis of the left arm—an evidence of a more serious lesion in the brain and nerve centre. I stated to the parents my apprehensions of the existence of this serious lesion; while exhibiting to them the rent in the visual organs, which till now they were not aware of.

With a view to palliate and sustain for a time the already sinking energies, I prescribed calomel, 1 gr.; quinine, 2 grs.; every four hours, till six doses were given, provided the patient could be had to swallow: and ordered lead-water and laudanum to be applied to the eyes; blisters to the temples; volatile liniment to the back of the neck and along the spine; with cold water to the head, whenever the surface was preternaturally hot. This treatment was persevered in with such alterations as the condition of the patient seemed to suggest from time to time, but all to no permanent good. The little unfortunate sufferer continued gradually to sink into a deep and deeper lethargy, which ended in a final cessation of all the functions on the fourth day from the convulsive struggle.

A few hours previous to death, the remaining portion of the aqueous humor of the left eye flowed out through the rent in the cornea.

I sought a *post-mortem* examination of the brain, but did not obtain permission, which I very much regret.

